# Genome Instability and Senescence Are Markers of Cornelia de Lange Syndrome Cells

**DOI:** 10.3390/cells13232025

**Published:** 2024-12-07

**Authors:** Maddalena Di Nardo, Ian D. Krantz, Antonio Musio

**Affiliations:** 1 Institute for Biomedical Technologies, National Research Council, 56124 Pisa, Italy; maddalena.dinardo@itb.cnr.it; 2 Roberts Individualized Medical Genetics Center, Division of Human Genetics, The Department of Pediatrics, The Children’s Hospital of Philadelphia and the Perelman School of Medicine at the University of Pennsylvania, Philadelphia, PA 19104-4318, USA; krantz@chop.edu

**Keywords:** Cornelia de Lange syndrome, cohesin, *NIPBL*, *HDAC8*, *SMC1A*, genome instability, senescence, oxidative stress

## Abstract

Cornelia de Lange syndrome (CdLS) is a rare, dominantly inherited multisystem developmental disorder. Pathogenic variants in genes encoding the structural subunits and regulatory proteins of the cohesin complex (*NIPBL*, *SMC1A*, *SMC3*, *HDAC8*, and *RAD21*) are the primary contributors to the pathogenesis of CdLS. Pathogenic variations in these genes disrupt normal cohesin function, leading to the syndrome’s diverse and complex clinical presentation. In this study, we discovered that cells harboring variants in the *NIPBL*, *SMC1A* and *HDAC8* genes exhibit spontaneous genome instability, elevated oxidative stress and premature cellular aging. These findings suggest that cohesin plays a critical role in maintaining proper cellular function and highlight its contribution to the pathophysiology seen in the related diagnoses.

## 1. Introduction

Cohesin forms a ring-shaped complex composed of four core structural members, SMC1A, SMC3, RAD21 and STAG1/2. SMC1A and SMC3, belonging to the SMC protein family, are long, flexible coiled-coil proteins forming a V-shaped heterodimer that dimerizes via the SMC hinge domains on one side and are connected by the kleisin RAD21 subunit at the globular ATP-head domains on the other side. In addition, RAD21 interacts via its middle region with STAG1/2 proteins, ensuring a stable association between chromatin and cohesin. The sister chromatid cohesion mediated by cohesin is indispensable for proper chromosome segregation during the cell cycle [1,2]. Cohesin also modulates chromatin organization to mediate long-range interactions, controls gene expression regulation and repairs damaged DNA by ensuring proximity of sister chromatids [3,4,5]. Pathogenic variants within or dysregulation of the genes that encode structural cohesin components or regulators have been associated with chromosome aneuploidy, chromosomal instability and DNA damage repair errors, which are common features of cancer [4]. In fact, somatic variants and altered expression have been identified in Ewing’s sarcoma, glioblastoma, colorectal carcinoma, breast cancer, lung carcinoma, urothelial bladder carcinoma, myeloid neoplasms and melanoma [6,7,8,9,10,11,12,13,14,15]. Germline pathogenic variants of cohesin genes result in a group of human developmental diagnoses collectively called “disorders of transcriptional regulation (DTRs)” [16]. Of the DTRs, Cornelia de Lange syndrome (CdLS, OMIM #122470, #300590, #610759, #30882 and #614701) occurs most frequently, with an incidence of between 1:10,000 and 1:30,000 live births. CdLS is a sporadic and genetically heterogeneous autosomal or X-linked disease affecting multiple organs and systems. Affected individuals are characterized by pre- and post-natal growth delay, intellectual disability, limb differences and characteristic facial features [17]. CdLS is caused by pathogenic variants in the *NIPBL*, *SMC1A*, *SMC3*, *RAD21* and *HDAC8* genes [18,19,20,21]. The molecular mechanisms of CdLS are not well understood. CdLS cell lines and developmental models of CdLS, such as mice and zebrafish, show modest, wide-ranging and conserved perturbations in gene expression [22,23,24,25,26]. It is thought that defective loop extrusion mediated by cohesin provokes an alteration in the chromatin structure, leading to gene dysregulation [27]. However, gene expression dysregulation is not the only marker of CdLS cells. Mutations in cohesin also impair the cell’s ability to repair damaged DNA [28,29]. Consequently, cells from individuals with CdLS can exhibit spontaneous chromosome anomalies [30]. These cells also show increased sensitivity to the interstrand cross-linking agent mitomycin C (MMC). Moreover, when exposed to X-rays during the G2 phase of the cell cycle, where repair processes rely on the establishment of sister chromatid cohesion, there is a marked dose-dependent increase in the formation of sister chromatid exchanges (SCEs) and double-strand breaks (DSBs). This finding indicates that CdLS cells manifest as defective in homologous recombination (HR) DNA repair, leading to delayed and/or faulty repair of damaged DNA [29,31,32,33]. We showed that two CdLS cell lines carrying variants in the *SMC1A* gene are characterized by high levels of oxidative stress, genome instability and reduced cell lifespan [34]. Until now, no systematic study had been performed to investigate whether genome instability and senescence occurrs in CdLS cells, beyond *SMC1A*-mutated cells. To gain insight into this topic, we cultured CdLS cell lines harboring pathogenic variants of the *HDAC8* and *NIPBL* genes. Here, we found that CdLS-derived cell lines exhibited increased oxidative stress, spontaneous genome instability, and premature cellular aging, features that can be considered as biomarkers for CdLS. These observations underscore the crucial role of cohesin in preserving cellular integrity and highlight its significant impact on CdLS pathogenesis.

## 2. Materials and Methods

### 2.1. Cell Culture

Primary human fibroblasts were grown in RPMI 1640 medium supplemented with 10% fetal bovine serum, 1% L-glutamine and antibiotics (100 U/mL penicillin, 0.1 mg/mL streptomycin) in a humidified 5% CO_2_ atmosphere. We used six CdLS fibroblast cell lines derived from patients carrying variants in the *HDAC8* and *NIPBL* genes. In addition, a previously analyzed *SMC1A*-mutated cell line [34] was used as a positive control. Two cell lines purchased from Coriell Cell Repositories and a homegrown cell line called NIG served as control samples. All cell lines were cultured in duplicate. During the early passages, cells were subcultured on a weekly basis once the monolayer became confluent. If the cells did not reach confluency within one week, the culture was classified as “senescent”, and fresh medium was replenished weekly. After a period of four weeks, if confluency was still not achieved, the culture was deemed to have fully “senesced”.

### 2.2. Ethics Statement

This study was conducted according to the principles expressed in the Declaration of Helsinki. All patients were enrolled under an IRB-approved protocol of informed consent at The Children’s Hospital of Philadelphia and by the Children Ethics Committee (CEP, protocol number 130/2016).

### 2.3. Treatments

X-ray sensitivity was assayed by exposing cells to 2 Gy using a linear accelerator with a 6 MV photon energy source. For the drug sensitivity assay, fibroblast cell lines were seeded 24 h prior to treatment with 1, 2, 3 and 4 μM MMC for 1 h, followed by washing and recovery for 6 days.

### 2.4. Cytogenetic Analysis

KaryoMax Colcemid (ThermoFisher, Waltham, MA, USA) was introduced to the cell cultures for 90 min, after which the cells were incubated for 30 min in a hypotonic solution (0.075 M KCl) at 37 °C. The cells then underwent several washes with Carnoy’s fixative (methanol:acid, 3:1). Following fixation, 100 metaphases were stained with Giemsa and directly examined using a Leica DM2500 microscope. Gaps and breaks were scored according to the criteria laid down by ISCN 1985 as follows: a gap is a non-staining (achromatic lesion) region (of a single chromatid or at the same locus in both chromatids of a chromosome in cases of a chromatid gap or chromosome gap, respectively) and a break is a discontinuity in which there is a clear misalignment (of one of the chromatids or at the same locus in both chromatids in cases of a chromatid gap or chromosome gap, respectively) [35].

### 2.5. Immunofluorescence Labeling and Microscopy

To examine γ-H2AX foci formation following irradiation, cells were fixed in 2% paraformaldehyde for 10 min, permeabilized in 0.2% Triton X-100 and blocked in PBS with 1% BSA. Thereafter, cells were incubated with a primary antibody against γ-H2AX (Trevigen, Minneapolis, MN, USA) for 1 h at 37 °C in a humidified atmosphere and then incubated with Alexa Fluor 488-conjugated goat anti-rabbit secondary antibody (Molecular Probes, Eugene, OR, USA). After three washes with PBS/0.2% Triton X-100, the cells were counterstained with 4′,6-diamino-2-phenylindole (DAPI) in mounting medium (Vector Laboratories, Newark, CA, USA). γ-H2AX foci were scored manually from 300 cells from three independent experiments using a Leica DM2500 microscope.

### 2.6. Senescence-Associated β-Galactosidase (SA-β-gal) Staining

SA-β-Gal staining was performed using the Senescence Cell Histochemical Staining Kit (Sigma Aldrich, St. Louis, MO, USA) following the manufacturer’s protocol. Briefly, fibroblasts were washed three times with PBS and fixed with paraformaldehyde for 10 min. After washing, fixed fibroblasts were incubated in SA-β-Gal staining solution at 37 °C. Stained cells were imaged using a Leica DM2500 microscope. Total fibroblasts and SA-β-gal positive fibroblasts were counted in three random fields per dish.

### 2.7. Oxidative Stress

The level of oxidative stress was measured by protein carbonyl content with the enzyme-linked immunosorbent assay (ELISA) using a Protein Carbonyl ELISA Kit (Cell Biolabs, San Diego, CA, USA) as previously described [34].

### 2.8. RNA Purification and Quantitative Real-Time PCR (qRT-PCR) Analysis

cDNAs were prepared with SuperScript II reverse transcriptase using oligo dT (Invitrogen; Waltham, MA, USA) from total RNA (RNAeasy Mini-kit, Qiagen, Hilde, Germany). qRT-PCR analyses were performed in triplicate using the Rotor Gene 3000 (Corbett, Manchester, UK). Expression was normalized with respect to the mean level of expression of the *β-Actin* housekeeping gene. Since no difference was found in control cell lines, data were pooled. The primers used for mRNA expression analysis are listed in Supplementary Table S1.

### 2.9. Statistics

Data were analyzed by Student’s *t*-test. *p*-values of <0.05 were considered statistically significant.

## 3. Results

### 3.1. CdLS Cells Display Reduced In Vitro Lifespan

Patients with CdLS exhibit signs of premature aging, including symptoms such as rumination, cutis verticis gyrata, and early-onset osteoporosis, these often appearing during their teenage years. Additionally, these individuals may experience premature graying of hair and noticeable changes in the skin, particularly on the face, leading to an appearance that is more aged than expected for their chronological age [36]. This notion was further supported by the observation that *SMC1A*-mutated cell lines showed a reduced in vitro lifespan, suggesting that premature aging and a shortened cell life are intrinsically linked [34]. To determine whether this is a phenomenon common in CdLS independent of causative genes, we analyzed six CdLS primary fibroblast cell lines carrying variants in the *HDAC8* and *NIPBL* genes. The *HDAC8*-mutated cells harbor two different pathogenic missense variants (539A>G and 1001A>G, leading to H180R and H334R amino acid changes, respectively). These variants severely affect the HDAC8 deacetylase activity. Of the *NIPBL*-mutated cells, three are characterized by premature stop codons caused by nonsense or frameshift variants (1372C>T, 2479_2480delAG leading to Q458X and R827gfsX2 amino acid changes, respectively) and one carries a missense variant (6893G>A leading to R2298H change). This latter variant maps to the HEAT domain and likely alters the interaction of NIPBL with other proteins. In addition, a cell line with a missense variant (c.3146G>A leading to p.R1049Q change) in the *SMC1A* gene was used as a positive control (Table 1). This variant is located at a coiled-coil domain. It is thought that coiled-coil interactions are important for the correct folding of an SMC monomer and are thus crucial for the formation of the head domain.

**Figure 1:**
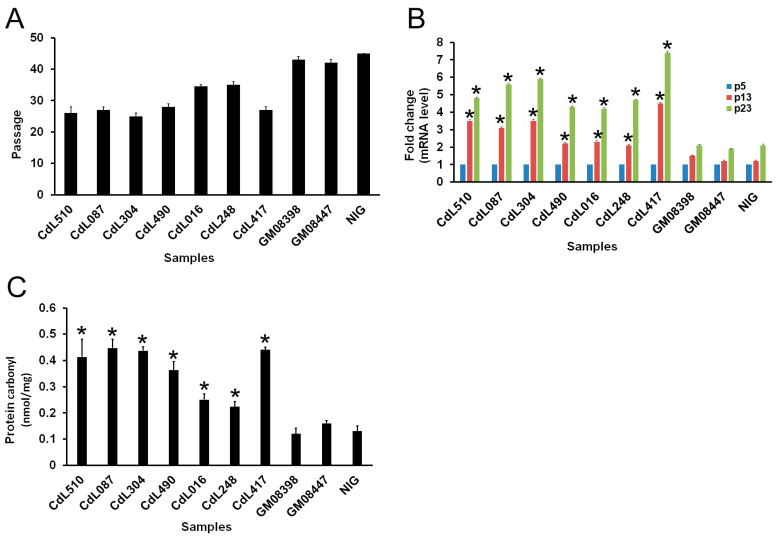
CdLS cell markers during in vitro cell culture progression. (**A**) *NIPBL*-, *HDAC8*- and *SMC1A*-mutated cells are characterized by replicative senescence. (**B**) RT-qPCR analysis of p16 at three different passages in vitro. Data represented the average ± SE from three independent experiments. (**C**) Protein carbonyl content, as a marker of oxidative stress, was measured in *NIPBL*-, *HDAC8*- and *SMC1A*-mutated cells. * *p* < 0.05.

Cell culture senescence was defined as the number of cell population doublings at which the cell culture failed to reach confluence at 1 week after subcultivation. By this definition, CdLS cell cultures reached their senescent phase at lower in vitro passages than control cells. CdLS cells became senescent between the 25th and the 34.5th passage, with a considerable decrease (about 45% in NIPBL cells carrying premature stop codon) in their *in vitro* lifespan when compared with control cells (Figure 1A and Supplementary Table S2).

β-Galactoside staining, a measure of cell senescence, showed that the senescence ratio of CdLS fibroblasts had increased considerably. The results demonstrated that the percentage of the SA-β-gal-positive senescent cells increased significantly during in vitro cell culture progression, ranging from 25% to 88%. The RT-qPCR results showed elevated mRNA expression levels of the senescence markers, CDKN2A p16 (cyclin-dependent kinase inhibitor 2A) and CDKN1A p21 (cyclin-dependent kinase inhibitor 1A) as compared to control cells (Figure 1B, *p* < 0.05, Supplementary Figure S1). Next, we analyzed the level of carbonyl derivatives of proline, lysine, arginine and threonine residues as a consequence of protein oxidation during cell progression. These results indicated that the levels of protein carbonyl remained comparatively low in control cells but increased in CdLS cells (Figure 1C). Overall, these findings suggest that CdLS cells exhibit a shortened lifespan in vitro that is associated with high levels of oxidative stress.

### 3.2. CdLS-Causative Genes Are Associated with Increased Sensitivity to Genotoxic Agents

**Figure 2:**
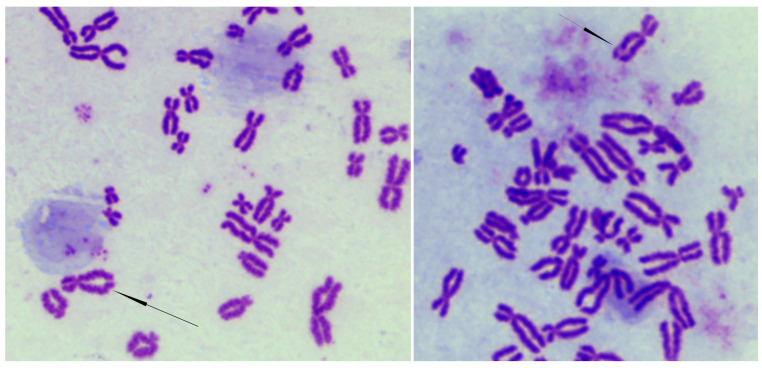
CdLS and damaged DNA repair during in vitro cell culture progression. Partial Giemsa-stained metaphases showing a chromatid break (indicated by an arrow) and a chromatid gap (indicated by an arrow) during *in vitro* cell culture progression of CdL510 (**left**) and CdL248 (**right**) cells, respectively. According to ISCN 1985, the break is clearly visible as region in which there is a misalignment of one of the chromatids.

Since cohesin is involved in DNA repair and maintaining genome stability [4], we investigated the occurrence of spontaneous genomic instability and whether CdLS cells are sensitive to DNA-damaging agents. At passage 13, the incidence of spontaneous chromosome abnormalities was observed to be significantly higher in CdLS cell lines as compared to control cell lines (Figure 2 and Table 2).

In particular, cells carrying a *NIPBL*-frameshift variant showed more aberrations (ranging from 11 to 15 in one hundred metaphases) than cells with the *NIPBL*-missense (7–9 in one hundred metaphases) or *HDAC8*-missense variants (ranging from 6 to 7 in one hundred metaphases). However, the *SMC1A*-mutated cell line displayed the highest frequency of chromosome aberrations, 23-25 in one hundred metaphases.

**Figure 3:**
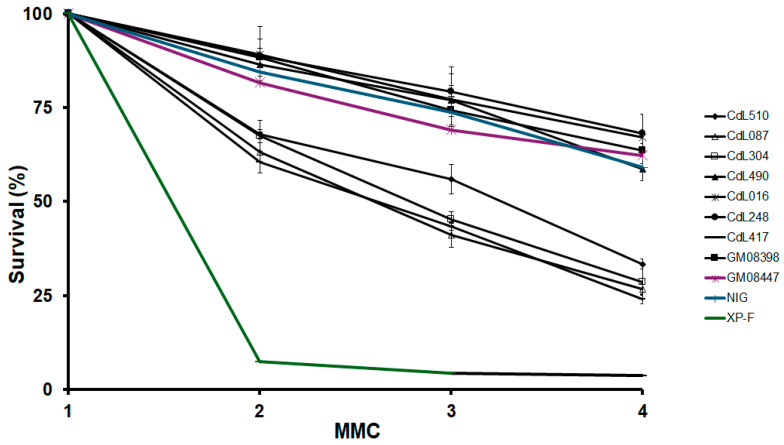
Sensitivity of cohesin-mutated cell lines to MMC at passage 13 of *in vitro* cell culture. Control fibroblasts, a Xeroderma Pigmentosum cell line (XP-F) and cell lines carrying variants in the *HDAC8*, *NIPBL* and *SMC1A* genes were plated the day before the exposure. Cells were treated with different doses of MMC (1, 2, 3 and 4 μM) for 1 h. The survival was evaluated after 6 days of recovery. The data represent the average of three independent experiments.

Then, we exposed cohesin-mutated cell lines to MMC, a well-characterized DNA crosslinking agent. In this analysis, a consistent response was observed, with *SMC1A*-mutated cells and all cell lines carrying nonsense or frameshift *NIPBL* variants displaying heightened sensitivity to MMC compared to the control cells. However, their sensitivity can be categorized as moderate, as it did not reach the sensitivity levels observed in a Xeroderma Pigmentosum (XP-F) cell line. Instead, all cell lines with missense variants in the *NIPBL* and *HDAC8* genes showed similar sensitivity to control cells (Figure 3).

**Figure 4:**
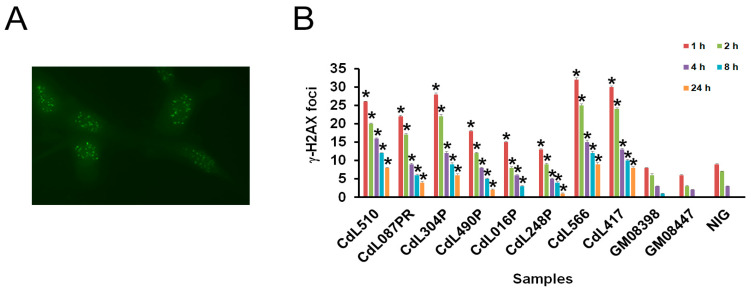
(**A**) γ-H2AX foci after 30 min following 2 Gy irradiation in *NIPBL*-mutated cells (CdL510 cell line). (**B**) Time course of γ-H2AX foci disappearance following 2 Gy irradiation. Error bars represent the SE from the analysis of 300 cells from three independent experiments. * *p* < 0.05.

To further assess the occurrence of chromosomal instability, we examined γ-H2AX foci formation. Our results showed that CdLS cells exhibited a higher number of γ-H2AX foci 30 min after exposure to 2 Gy of irradiation compared to control cells. The average number of γ-H2AX foci per cell ranged from 46 in *NIPBL*-mutated cells to 28 in cells harboring *HDAC8* variants (Figure 4A and Supplementary Figure S2).

Once again, cells with *NIPBL* variants resulting in a premature stop codon and those with a missense variant in the *SMC1A* gene showed a considerable increase in the number of foci. In particular, cells with the *SMC1A* variant demonstrated the highest level of γ-H2AX foci, an average of 51/cell. Time course experiments revealed that 4 h after irradiation, about 50% of DSBs were repaired in both the control and CdLS cells. Thereafter, it declined almost to the control level. For repair times of 8 h and 24 h, both cells carrying *NIPBL* frameshift variants and cells with the *SMC1A* variant showed more foci than cells with the *HDAC8-* and *NIPBL*-missense variants (Figure 4B). These data suggest that genome instability and an impaired DNA repair pathway may be a common mechanism in CdLS-derived cell lines.

## 4. Discussion

CdLS is a genetic multisystem developmental disorder. Variants in the *NIPBL* gene account for approximately 60% of CdLS cases, while a smaller proportion of affected individuals, comprising 5–7% of cases, have pathogenic variants in the *HDAC8*, *RAD21*, *SMC1A* and *SMC3* genes [37]. CdLS is characterized by a wide range of phenotypic effects, including small size, craniofacial and limb differences, multiorgan defects, intellectual disability and signs of premature aging relative to their chronological age [17]. Although the functional consequences of cohesin disruption in CdLS remain incompletely understood, we have previously shown that variants in both *SMC1A* and *SMC3* genes make these cells more susceptible to protein oxidation, DNA damage and cellular senescence [34,38]. Here, we show that cells carrying pathogenic variants in the *HDAC8* and *NIPBL* genes also display genomic instability, sensitivity to genotoxic agents and premature in vitro senescence, suggesting that they are markers of CdLS cells irrespective of the causative genes.

The mean of spontaneous aberrations per cell ranges from 6 to 7 and from 7 to 15 in *HDAC8* and *NIPBL*-mutated cells, respectively. It is interesting to note that cells with *NIPBL* variants leading to a premature stop codon have approximately twice the number of aberrations compared to those with missense variants in the *NIPBL* and *HDAC8* genes. However, these values are significantly lower than the number of aberrations present in cells with the *SMC1A* variant. This could reflect the different roles of *NIPBL*, *HDAC8* and *SMC1A* in preserving genome stability through cohesin functions. NIPBL, in association with its molecular partner MAU2, is involved in cohesin deposition onto chromatin. The silencing of *NIPBL* increases cellular sensitivity to genotoxic agents, and the repair of DSBs caused by endonuclease cleavage and laser microirradiation requires the cohesin complex to accumulate at the sites of damage, a process reliant on the NIPBL–MAU2 loading complex [39,40,41]. Conversely, the deacetylation of cohesin by the deacetylase HDAC8 is essential for the recycling of cohesin complexes, enabling them to participate in subsequent rounds of sister chromatid cohesion [20,42]. The loss of HDAC8 activity leads to the elevated acetylation of SMC3, which is reloaded onto chromatin, leading to a reduced occupancy of cohesin at its localization sites [42]. It is likely that this results in a distinct DNA repair pattern alteration to be observed in CdLS cell lines harboring mutations in either *NIPBL* or *HDAC8*. SMC1A is a member of the cohesin core. Following DNA damage, SMC1A is phosphorylated by ATM or ATR kinases on the serine 957 and serine 966 residues. Cells expressing mutated SMC1A that cannot undergo this phosphorylation display impaired DNA repair mechanisms and reduced cell viability [42,43,44,45]. This indicates that SMC1A is directly involved in preserving the genome stability of human cells, ensuring an effective and coordinated response to repair DNA damage. This is supported by the finding that *SMC1A* mutations have been identified in human cancers characterized by genome instability, such as colorectal cancer [15,46,47,48,49].

Though CdLS cells exhibit genome instability, it has been shown there is no increased risk of cancer in CdLS patients, although *NIPBL* variants may genetically predispose to early Barrett’s esophagus development [50]. Since tumorigenesis develops over many years because of the accumulation of specific variants, it is likely that additional genetic alterations are required for a fully malignant transformation beyond the initial cohesin variants.

Different pathogenic variants in the *HDAC8*, *NIPBL* and *SMC1A* genes are associated with varying frequencies of chromosomal aberrations. In fact, cells with a mutation in the *SMC1A* gene exhibited the highest level of chromosomal aberrations, followed by cells with nonsense or frameshift mutations in *NIPBL* and, finally, cells with missense mutations in *NIPBL* and *HDAC8*. In addition, a clonogenic survival assay revealed that *SMC1A*-mutated cells (CdL417) and all cell lines (CdL510, CdL087 and CdL304) harboring *NIPBL* variants leading to premature stop codon displayed an increased sensitivity to MMC. These observations suggest that both *SMC1A* and *NIPBL* may play a role in the resolution of interstrand cross-linking. Therefore, we propose that these cytogenetic assays can be used to differentiate between CdLS patients with variants in different causative genes. Chromosomal breakage assays are used as a diagnostic tool for other genetic diagnoses. For example, Fanconi anemia (FA), an autosomal recessive diagnosis marked by pancytopenia, diverse congenital anomalies, heightened cancer susceptibility and chromosomal instability in cultured cells with an increased vulnerability to chromosomal breakage when exposed to a non-toxic concentration of the bifunctional alkylating agent diepoxybutane [51]. The ability to distinguish between the different CdLS-causative genes in an *in vitro* system that measures the occurrence of spontaneous chromosome aberrations may represent a test that can help differentiate genetic subtypes of CdLS and could help in stratifying patients in which a known molecular etiology has not been identified. Additionally, this assay may be able to serve as a biomarker to assess the efficacy of various therapeutic modalities and drug screening in cellular assays.

By employing γ-H2AX foci analysis to assess DSB repair following irradiation, we show that mutations in *SMC1A*, *NIPBL* and, to a lesser extent, *HDAC8* cause defects in DNA repair in primary fibroblasts. Although *SMC1A*- and *NIPBL*-mutated cells repair most DSBs in a manner similar to *HDAC8*-mutated and control cells, a small subset of irradiation-induced breaks remains unresolved. It is reasonable to infer that DNA repair efficacy may depend on the complexity of the damage at individual sites and the specific variants that disrupt these repair mechanisms, leading to persistent γ-H2AX foci indicative of permanent DNA damage.

Cellular senescence is a biological process in which cells permanently stop dividing but do not die. This state is often a response to various forms of stress, such as DNA damage, oxidative stress or telomere shortening, and plays a crucial role in aging, tumor suppression and tissue repair.

We observed that CdLS cells with *NIPBL* frameshift variants entered senescence at the 26th or 27th passage, showing a significant decrease in their lifespan compared to three control cell lines. This senescence was further validated using a β-galactosidase assay, where the percentage of positive cells ranged from 78% to 82% in control cells and was approximately 90% in CdLS cells. Results supportive of this included RT-qPCR data, which indicated that the mRNA expression levels of the senescence markers p16 and p21 were elevated. It is probable that both persistent DNA damage and premature aging contribute significantly to the pathogenesis of CdLS. These factors may intersect in influencing the development and progression of the syndrome, highlighting the complex interplay between genomic instability and accelerated cellular aging observed in individuals with CdLS. This notion is further supported by the observations that senescence is exacerbated by persistent DNA damage and the high levels of γ-H2AX foci that are observed in the *Nipbl*- and *Hdac8*-mutant mice [52].

## 5. Conclusions

In conclusion, our data indicate that genome instability and senescence serve as distinctive markers of CdLS cells. These cellular characteristics underscore the pathophysiological complexities of CdLS, providing crucial insights into the molecular consequences of cohesin disruption and the impact that this has on the phenotype and outcomes in related diagnoses. The presence of genome instability and premature senescence highlights the multifaceted nature of CdLS, where abnormalities in the cellular processes contribute to the diverse clinical manifestations observed in affected individuals.

## Figures and Tables

**Table 1:** Human primary fibroblast cell lines used in this work.

Cell Line	Gene	Gene Variation	Amino Acid Change
CdL510	NIPBL	c.1372 C>T	p.Q458X
CdL087	NIPBL	c.2479_2480del AG	p.R827gfsX2
CdL304	NIPBL	c.2479_2480del AG	p.R827gfsX2
CdL490	NIPBL	c.6893G>A	p.R2298H
CdL016	HDAC8	c.539A>G	p.H180R
CdL248	HDAC8	c.1001A>G	p.H334R
CdL417	SMC1A	c.3146G>A	p.R1049Q
GM08398	Control		
GM08447	Control		
NIG	Control		

**Table 2:** Spontaneous chromosome aberrations at passage 13 of *in vitro* cell culture.

Cell Line	Chromosome Aberrations	Metaphases
CdL510a	15	100
CdL510b	13	100
CdL087a	12	100
CdL087b	14	100
CdL304a	13	100
CdL304b	11	100
CdL490a	9	100
CdL490b	7	100
CdL016a	7	100
CdL016b	6	100
CdL248a	6	100
CdL248b	6	100
CdL417a	23	100
CdL417b	25	100
GM08398a	3	100
GM08398b	4	100
GM08447a	2	100
GM08447b	4	100
NIGa	2	100
NIGb	3	100

## Data Availability

The relevant data supporting the findings of this study are available in this article and its Supplementary Information files.

## Supplementary Materials

The following supporting information can be downloaded at: https://www.mdpi.com/article/10.3390/cells13232025/s1, Table S1: Primer sequences used in this study. Table S2: Cellular passage at which cells become senescent in vitro. Figure S1: RT-qPCR analysis of p16 at three different passages in vitro. Data represent the average from three independent experiments. Figure S2: γ-H2AX foci 30 min after exposure to 2 Gy.
