# Supplementary material for: Genome Instability and Senescence Are Markers of Cornelia de Lange Syndrome Cells
Source: Cells. 2024 Dec 7;13(23):2025. doi: 10.3390/cells13232025 (PMC11640591; doi:10.3390/cells13232025)
Supplement: Supplementary file 1 [file cells-13-02025-s001.zip › cells-3312394-supplementary.pdf]

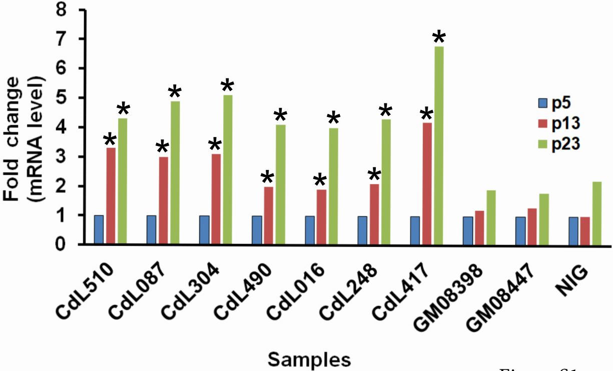
Fold change
(mRNA level)
0
1
2
3
4
5
6
7
8
p5
p13
p23
CdL510
CdL087
CdL304
CdL490
CdL016
CdL248
CdL417
GM08398
GM08447
NIG
Samples

Figure S1

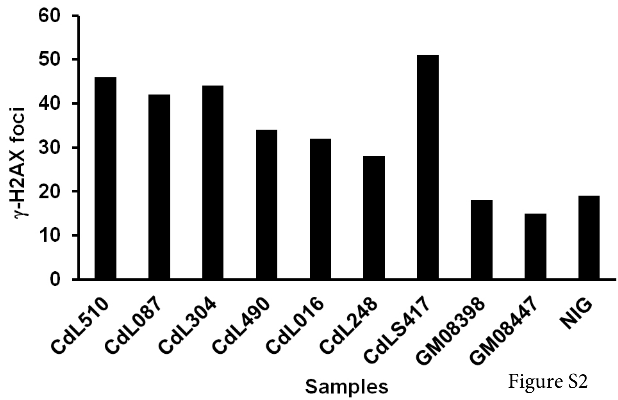
60
50
40
30
20
10
0
γ-H2AX foci
CdL510
CdL087
CdL304
CdL490
CdL016
CdL248
CdLS417
GM08398
GM08447
NIG
Samples

Figure S2

Supplementary Table S1. Primer sequences used in this study

**Gene name**	**F/R**	**Sequences 5'-3'**
p16	F	GAGGACCCCACCACCCTCTC
	R	ATACCGCAAATACCGCACGA
p21	F	CTTTCTTTGTGTATTTGCCA
	R	AACTCCTGAGCCTGTTTCGT
β-actin	F	TGCTATGTTGCCCTAGACTTCG
	R	GTTGGCATAGAGGTCTTTACGG

Supplementary Table S2. Cellular passage at which cells become senescent in vitro.

Cell line	Culture 1 (passage)	Culture 2 (passage)
CdL510	28	24
CdL087	28	26
CdL304	24	26
CdL490	29	27
CdL016	35	34
CdL248	36	34
CdL417	26	28
GM08398	44	42
GM08447	41	43
NIG	45	45
